# Effect of problem-based learning on severity classification agreement by triage nurses

**DOI:** 10.1186/s12912-021-00781-2

**Published:** 2021-12-20

**Authors:** Kyeongmin Jang, Eunmi Jo, Kyoung Jun Song

**Affiliations:** 1 Department of Nursing, Bucheon University, 56, Sosa-ro, Bucheon-si, Gyeonggi-do Republic of Korea; 2 Department of Nursing, Seoul Metropolitan Government-Seoul National University Boramae Medical Center, 20 Boramae-ro 5-gil, Dongjak-gu, 07061 Seoul, Korea; 3 Department of Emergency Medicine, Seoul Metropolitan Government-Seoul National University Boramae Medical Center, Seoul National University College of Emergency Medicine, Seoul, Korea

**Keywords:** Triage, Problem-based learning, Length of stay, Emergency department, Patient safety

## Abstract

**Background:**

Differences in the classification results among triage nurses in the emergency room can be improved by training or applying an algorithm. This study aimed to confirm whether the agreement among triage nurses could be improved through learner-led problem-based learning.

**Methods:**

This study had a single-group time series design to investigate the effect of problem-based learning led by triage nurses on the agreement of Korean Triage and Acuity Scale classification results for patients who visited the emergency department. We extracted 300 patients each in May and August 2018 before learning began and 300 patients each in May and August 2019 after learning.

**Results:**

After problem-based learning was applied, the self-efficacy of triage nurses for emergency patient classification increased statistically significantly compared to before learning (7.88 ± 0.96, *p* < .001), and the weighted kappa coefficient was also found to be almost perfectly agreement (0.835, *p* < .001).

**Conclusions:**

In this study, problem-based learning improved the inter-rater agreement of Korean Triage and Acuity Scale classification results and self-efficacy of triage nurses. Therefore, problem-based learning can contribute to patient safety in the emergency department by enhancing the expertise of triage nurses and increasing the accuracy of triage classification.

## Background

According to statistics released by the National Emergency Medical Center in Korea in 2019, more than 10 million patients visited the emergency department (ED) during 2018, which was more than 20% higher than that in 2008 as a result, ED overcrowding is increasing [1]. As the number of patients who visited the ED increases, ED overcrowding has been pointed out as one of the most important problems [2]. One of the most important ways to relieve ED overcrowding is by reducing the length of stay of emergency patients [3, 4]. The ED stay time can be reduced by appropriately classifying the severity of emergency through accurate initial assessment of patients who visit the ED [5, 6].

Classifying the severity appropriately through initial assessment of patients visiting the ED is a process of determining where to receive treatment [7]. Accurate patient classification is essential for identifying patients who do not require prior intervention and optimizing emergency medical resources for immediate treatment [8]. Therefore, triage nurses in charge of initial assessment in the ED should quickly and appropriately categorize the severity of patients visiting the ED. This is one of the most important tasks of ED triage nurses [9].

For initial assessment and classification tool of emergency patients in Korea, various classification tools were used in each hospital until recently [10]. In 2012, the Korean Triage and Acuity Scale (KTAS) Committee affiliated with the Korean Society of Emergency Medicine developed KTAS as a Korean type emergency patient classification tool based on Canadian Triage and Acuity Scale (CTAS), Canada’s five-stage classification tool, by modifying and supplementing it to suit the domestic situation [11]. Since 2016, the law on emergency medical care has been changed. All EDs have been using the KTAS to classify patients visiting the ED [12]. This severity classification is performed by nurses in the United States [12]. In most EDs in Korea, nurses perform patient classification through initial evaluation. However, there is a difference in the degree of agreement among triage nurses according to ED experience or clinical experience of nurses who perform the evaluation. Objective evaluation may be difficult in psychological and mental health areas, although respiratory diseases could be objectively evaluated [9]. In addition, the severity classification may be different because different factors determine the degree of urgency [13]. Even if there are clear criteria for classification, there might be differences in the severity classification results among evaluators. In one study determining the agreement between nurses and emergency doctors [14], the Kappa coefficient was 0.659. In addition, there was a significant difference in classification between male and female nurses (*p* = .003) [14]. Another study has assessed the degree of agreement among nurses [15] and found that the kappa coefficient is 0.79. The kappa coefficient was 0.721 in one study that determined the degree of agreement between nurses and medical students [16]. In one study that determined the agreement between triage nurses and an expert group consisting of KTAS instructors, the weighted kappa coefficient was 0.77 [17]. These results are interpreted by Landis & Koch, which shows a ‘substantial agreement’, but there are some differences among evaluators [18]. As such, differences in severity classification results among triage nurses may occur, and the differences in classification results may cause patient dissatisfaction and threaten patient safety. The agreement of these classification results can be improved by training or applying an algorithm [13].

Problem-based learning (PBL) is a small group of learning method that enables students to present problems and learn knowledge, skills, and attitudes to solve problems [19]. This is a learning method that can develop not only knowledge acquisition, but also reasoning ability and critical thinking ability [19]. In previous studies, after PBL was conducted, learning attitude [20, 21], critical thinking skill [22, 23], problem solving [21, 24], and knowledge [25, 26] of nursing students and medical students were improved. As such, it is believed that PBL, led by nurses, will help resolve the discrepancy in the severity classification results of ED patients and help improve the patient classification algorithm. However, previous studies that suggested a method to improve the agreement of the severity classification results through PBL were insufficient. It is necessary to confirm whether PBL led by triage nurses using actual patient cases helps to improve the agreement of classification results among triage nurses. Therefore, the purpose of this study is to investigate the effect of PBL led by a triage nurse on the consistency of KTAS classification results of patients who visited the ED.

## Methods

### Study design

This study had a single-group time series design to investigate the effect of PBL led by triage nurses on the agreement of KTAS classification results for patients who visited the ED.

### Study subjects

#### Patients

Bujang and Baharum have presented a minimum sample number of 396 with a significant level of 0.05, power of 90%, with a goal of 0.7 to 0.8 kappa coefficient for tools of grade 5 [27]. In another study that presented a monogram for calculating sample size with Kappa statistics, the required number of samples was at least 283 at significant level of 0.05, power of 80%, and kappa coefficient of 0.9 [28]. Therefore, this study included approximately 1,200 patients (600 patients before PBL and 600 patients after PBL, considering daily visits to the ED. Patients aged 15 years or older who had visited an ED during May and August 2018 for preliminary surveys and May and August 2019 for postmortem surveys respectively were included. Those who had insufficient initial evaluation nursing record in the electronic medical record were excluded.

#### Triage nurses

Subjects were nurses with KTAS certification in the ED who were in charge of initial patient classification during the study. A total of 51 nurses are working in the ED of the hospital in which this study was conducted, and one triage room is operated normally. Of a total of 51 nurses, about 15 nurses who have received the qualifications granted by the KTAS committee take turns classifying patients in the ED. We retrospectively investigated the results of triage nurses classifying patients who visited the ED. In May and August 2018, the preliminary survey period, 11 and 12 triage nurses were included, respectively, and in May and August 2019, the post-test period, 12 and 13 triage nurses were included, respectively.

### Study procedure

#### Problem based learning (PBL)

**Fig. 1:**
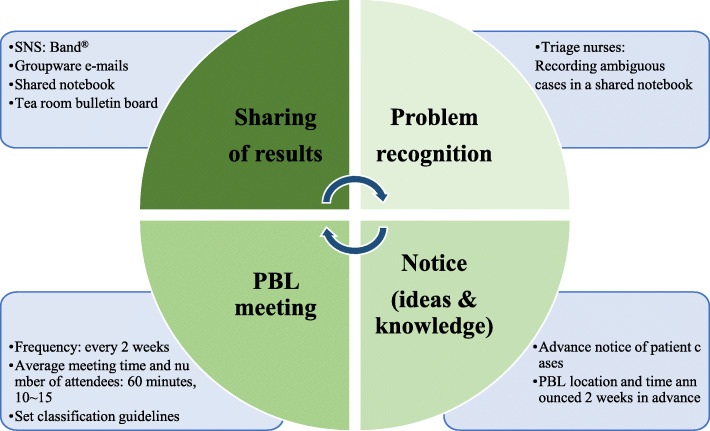
Problem based-learning procedure

**Fig. 2:**
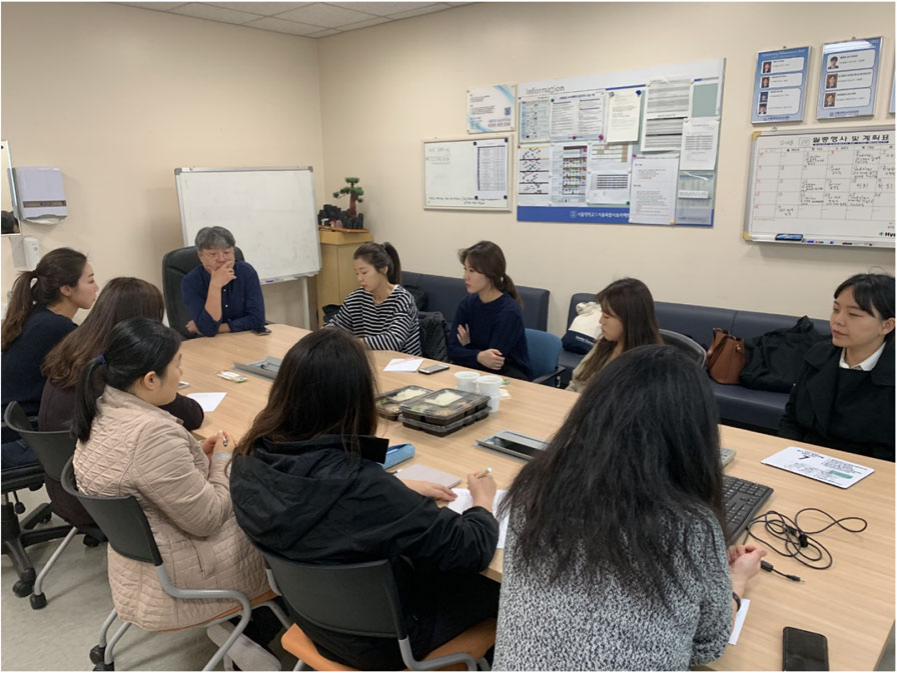
A scene from a PBL meeting with triage nurses and emergency physicians

PBL was led by triage nurses in charge of patient classification in the ED. Triage nurses in charge of patient classification in the emergency room were asked to record ambiguous cases during the initial patient classification task in a shared notebook placed in the triage room. In this learning meeting, triage nurses and emergency physicians gathered to present their opinions on the KTAS classification results of cases recorded in this shared note, and through discussions, a classification policy was set so that patients with similar symptoms can later visit the ED (Fig. 1). The date and time of PBL was announced two weeks in advance, and triage nurses were encouraged to participate voluntarily. When additional learning is needed for a specific case, one or two triage nurses reviewed the literature and made a presentation within 10 min at the next meeting, and the classification policy for the case was decided through further discussion. Social media, groupware e-mails, and shared notes in the triage room were used to share the decisions made in PBL with other ED nurses. This learning meeting lasted about 60 min each time, and about 10-15 people participated, and it was held twice a month (Fig. 2).

#### Data collection procedure

For pre-investigation, we extracted 300 patients using Microsoft’s Excel random function from EDs in May and August of 2018 before the PBL meeting began. We retrospectively reviewed emergency nursing information surveys stored in the electronic medical record (EMR). For post-investigation, we reviewed patient’s emergency nursing information surveys stored in the EMR for patients visiting the ED six months after the problem-based study meeting in May 2019 and August 2019 in the same way as the pre-investigation. Emergency nursing information surveys for patients who visited the ED during pre- and post- investigation periods were retrospectively reevaluated without knowing KTAS classification results evaluated by triage nurses by two Gold Standard nurses; one nurse was a KTAS instructor and a nurse with over 15 years of ED work experience, and the other nurse had over 15 years of emergency room work experience and over 10 years of triage work experience. We compared KTAS classification results classified by these Gold Standard nurses with those evaluated by triage nurses. We measured and compared the weighted Kappa coefficient, the coefficient of agreement.

### Study endpoints

#### Inter-rater agreement of KTAS

Gold Standard evaluators re-evaluated the KTAS classification results classified by triage nurses, and the weighted KAPPA coefficient was measured to confirm the degree of agreement between these two groups. Cohen suggested the Kappa result be interpreted as follows: values ≤ 0 as indicating no agreement and 0.01–0.20 as none to slight, 0.21–0.40 as fair, 0.41– 0.60 as moderate, 0.61–0.80 as substantial, and 0.81–1.00 as almost perfect agreement.

#### KTAS self-efficacy

The self-efficacy of the severity classification was investigated with the self-efficacy tool developed by Bandura [29]. This tool is a numeric evaluation scale, with 0 points for the leftmost and 100 points for the rightmost for self-efficacy for triage, and the subject selects one point on a continuous line. And scored. The higher the score, the higher the level of self-efficacy.

#### Length of ED stay

In the electronic medical record, length of ED stay (ED LoS) was defined as the time of discharge in the ED such as discharge or hospitalization in minutes from the time of receipt in the ED.

### Statistical analysis

Collected data were analyzed using SPSS for Windows 23.0 (IBM, Armonk, NY, USA). General characteristics of triage nurses and patients were subjected to descriptive statistics using nominal variables, frequency, and percentage. Continuous variables are presented as mean and standard deviation. One-way ANOVA was used to test the homogeneity of general characteristics of patients and triage nurses before and after PBL meeting. One-way ANOVA was used to compare ED stay time and self-efficacy of nurses before and after PBL. Weighted Kappa coefficient was measured to analyze the consistency of KTAS classification results between triage nurses and Gold standard evaluators.

### Ethical considerations

This study was conducted after receiving approval from the B Hospital Medical Research Ethics Review Committee, where researchers of this study were affiliated (IRB approval number: 20,190,426/30-2019-36/053).

## Results

### General characteristics of study patients and triage nurses according to investigation time

Homogeneity test was performed for general characteristics of study patients and triage nurses at the time of the investigation. As a result, the homogeneity of general characteristics according to the time of investigation was secured for both patients and triage nurses groups (Tables 1 and 2).

**Table 1:** General characteristics of study patients according to investigation time

Variables	2018. 05 (*n* = 300)	2018. 08 (*n* = 300)	2019. 05 (*n* = 300)	2019. 08 (*n* = 300)	F	*p*
Age (mean, SD)	58.1 (20.69)	56.4 (19.30)	55.69 (19.92)	58.5 (19.64)	1.32	0.266
Sex (male sex, %)	151 (50.3)	146 (48.7)	137 (45.7)	162 (54.2)		0.226
Mode of arrival (n, %)						
direct visit	264 (88)	273 (91)	278 (92.7)	264 (88)		0.174
outpatient	14 (4.7)	7 (2.3)	10 (3.3)	17 (5.7)		
via other hospital	22 (7.3)	18 (6)	11 (3.7)	19 (5.8)		
disease or trauma (disease, %)	248 (82.7)	231 (77)	228 (76)	243 (81)		0.138
Pain (yes, %)	145 (48.3)	154 (51.3)	147 (49)	136 (45.3)		0.532

*SD* standard deviation

**Table 2:** General characteristics of triage nurses according to investigation time

Variables	2018. 05 (*n* = 11)	2018. 08 (*n* = 12)	2019. 05 (*n* = 13)	2019. 08 (*n* = 12)	F	*p*
Age (mean, SD)	32.9 (5.16)	31.2 (4.98)	31.9 (5.18)	31.9 (5.00)	0.271	0.846
Sex (male sex, %)	5 (33.3)	4 (26.7)	3 (20.0)	3 (20.0)		0.691
Education (n, %)						
Diploma	1 (7.1)	1 (7.1)	1 (6.7)	1 (6.3)		0.942
Bachelor	10 (71.4)	9 (64.3)	12 (80.0)	13 (81.3)		
≥Master	3 (21.4)	4 (28.6)	2 (13.3)	2 (12.5)		
Total clinical career (mean, SD)	112.1 (59.75)	99.9 (56.26)	98.9 (54.35)	99.1 (53.62)	0.189	0.903
Career of ED (mean, SD)	91.4 (56.60)	85.7 (55.41)	98.9 (52.28)	87.2 (51.07)	0.063	0.992
Triage career (mean, SD)	24.0 (12.23)	24.3 (14.24)	23.07 (16.66)	23.88 (17.95)	0.017	0.997

*SD* standard deviation; *ED* emergency department

### ED LoS according to investigation time

Table 1 shows the ED LoS according to the time of investigation. Mean lengths of ED stay at the time of the investigation were 261.53 ± 215.74 min and 264.91 ± 201.63 min prior to the PBL in May 2018 and August 2018, respectively. Mean lengths of ED stay in May 2019 and September 2019 were 237.82 ± 164.13 min and 239.31 ± 181.21 min, respectively. After the PBL meeting, the mean ED LoS decreased, although such decrease was not statistically significant (*p* = .172) (Table 3).

**Table 3:** Length of ED stay according to investigation time

Investigation	ED LoS (Mean ± SD), min	F	*p*
2018. 05^a^	261.53 ± 215.74	1.67	0.172
2018. 08^b^	264.91± 201.63		
2019. 05^c^	237.82 ± 164.13		
2019. 08^d^	239.31 ± 181.21		

*ED* emergency department; *ED LoS* length of emergency department stay; *SD* standard deviation; ^a^pre-investigation 1 (2018. 05); ^b^pre-investigation 2 (2018. 08);^c^ post-investigation 1 (2019. 05); ^d^post-investigation 2 (2019. 08)

### Self-efficacy according to the time of investigation

Mean self-efficacy scores of KTAS classification at the time of the investigation of triage nurses were 6.50 ± 1.16 and 6.57 ± 0.94 in May 2018 and August 2018, respectively, before the PBL meeting was conducted. After the PBL meeting, mean self-efficacy scores in May 2019 and August 2019 were significantly increased to 7.73 ± 0.89 and 7.88 ± 0.96, respectively (*p* < .001) (Table 4). As a result of the post-HOC test, there was no statistically significant difference in self-efficacy between May 2018 and August 2018 before the PBL meeting. And there was no statistically significant difference in self-efficacy between May 2019 and August 2019 after the PBL meeting either. However, there were significant differences in self-efficacy between before and after PBL meeting (Table 4).

**Table 4:** Self-efficacy according to the time of investigation

Investigation	Self-efficacy (Mean ± SD)	F	*p*	Post-Hoc test
2018. 05_a_	6.50 ± 1.16	8.19	< 0.001	a, b < c, d
2018. 08^b^	6.57 ± 0.94			
2019. 05^c^	7.73 ± 0.89			
2019. 08^d^	7.88 ± 0.96			

*SD* standard deviation; ^a^pre-investigation 1 (2018. 05); ^b^pre-investigation 2 (2018. 08); ^c^post-investigation 1 (2019. 05); ^d^post-investigation 2 (2019. 08)

### Inter-rater agreement of KTAS classification results by investigation time

**Fig. 3:**
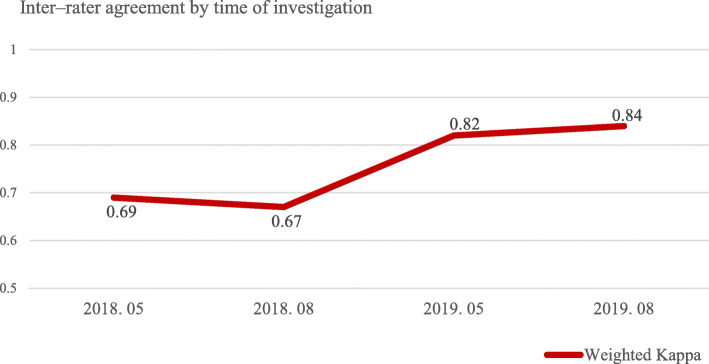
Inter-rater agreement by time of investigation

The inter-rater agreement of triage nurses in the ED showed weighted Kappa coefficients of 0.69 and 0.67 in May 2018 and August 2018, respectively, before the PBL meeting. However, after conducting the PBL meeting, showed weighted Kappa coefficients in May 2019 and August 2019 were 0.82 and 0.83, respectively, indicating ‘almost perfect’ agreement according to the interpretation proposed by Landis and Koch (1977). The inter-rater agreement of KTAS classification results between triage nurses and gold standard nurses was significantly (*p* < .001) improved after conducting PBL meeting (Fig. 3).

## Discussion

As Emergency Medical Service Act was amended, all EDs in Korea have been using KTAS to classify emergency patients since January 2016 [10]. This task is handled by nurses in most EDs. Many previous studies have reported that KTAS classification results might be different among evaluators. However, it is difficult to find studies suggesting a way to improve the inter-rater agreement among triage nurses. Therefore, this study attempted to improve the agreement among triage nurses’ self-efficacy for severity classification and inter-rater agreement among triage nurses through PBL meeting. Whether the improved agreement might affect the length of ED was also examined. Based on the results of this study, we would like to discuss ways to improve the professionalism of ED triage nurses.

### Self-efficacy for patient classification task

In this study, self-efficacy for emergency patient severity classification was improved after PBL led by ED triage nurses. This result cannot be directly compared to previous results because there is no previous research applying PBL. However, results of the study [30] were similar to results of the present study. They compared self-efficacy by developing and applying a web-based KTAS learning program with a control group [30]. Self-efficacy has been found to be improved after education compared to that before and after education in a study comparing HIRAID (History, Identify, Red flags, Assessment, Interventions, Diagnostics, reassessment and communication) framework developed to provide ED nurses with a structured and systematic approach to emergency patient assessment as a simulation training program [31]. The present study confirmed that the self-efficacy was improved through PBL led by triage nurses. However, previous studies have evaluated self-efficacy by applying web-based self-learning program or simulation program. We speculate that self-education, repetitive learning, and meetings to discuss learner-led clinical cases might have raised the self-efficacy of triage nurses’ KTAS classification.

CTAS and KTAS, unlike ESI which predicts and classifies emergency medical resources to be used, are tools that can classify patients based on their complaints [10]. In the first stage, patients are classified by age. In the second stage, patients are classified according to symptoms that patients complain. In the third stage, detailed symptoms included in the major classification are selected. In step 4, the KTAS level is determined by checking the pain or symptom of detailed symptoms selected in step 3. Finally, the place of treatment of the patient is determined according to the possibility of infectious disease that can transmit the disease [10]. Worldwide, emergency patients are classified by nurses in most EDs [9, 32]. Thus, KTAS classification results should be carefully classified because they can be directly linked to the calculation of emergency medical care fees. They are also important for the safety of patients. However, several previous studies have reported differences in classification among evaluators. The Kappa coefficient was 0.659 in a study of Rahmani et al. to determine the agreement of ESI classification between nurses and emergency physicians. In the study of Choi et al., classification was conducted by two research nurses for a total of 233 patients [15]. These patients were also reassessed by 10 triage nurses. They found that the weighted Kappa coefficient was 0.79. The weighted Kappa coefficient was 0.721 in a study of Kim et al., confirming the inter-rater agreement between nurses and medical students [16]. In addition, a weighted Kappa factor of 0.77 was reported in a study confirming the agreement between an expert group consisting of KTAS instructors and triage nurses for severity classification of ED pediatric patients [17]. The interpretation presented by Landis and Koch can be interpreted as a ‘substantial agreement’ [16]. However, there were some differences between the evaluators. In this study, the weighted Kappa coefficients were identified 0.687 and 0.67 respectively, before PBL meeting between triage nurses and Gold Standard nurses. After the PBL meeting was conducted, these weighted Kappa coefficients were improved significantly to 0.823 and 0.835, respectively, showing almost perfect agreement according to Landis and Koch’s interpretation [18]. Results of the present study could not be directly compared to results of previous studies because there is no such previous research. However, results of this study were similar to results of Rankin, Then, & Atack showing that online learning could help triage nurses improve their severity classification accuracy for emergency patients [33].

### Effect of PBL on the agreement of triage results

Barrows has proposed PBL method that can be conducted in small groups of student-led learning for medical students [19]. It is a learning method that learners can present their problems and learn knowledge and skills to solve problems through discussions in small groups so that they can develop reasoning skills and critical thinking skills to cope with given similar situations [19]. Previous studies have reported that learning attitude [20, 21], critical thinking skill [22, 23], problem solving ability [21, 24], and knowledge [25, 26] of nursing students and medical students are improved after the implementation of PBL. In the present study, learner-led PBL meeting was held with triage nurses working on the KTAS classification task to discuss cases that needed further discussion. In doing so, a consensus was reached and shared among ED nurses. These shared results might have helped their decision making for similar situations later and inter-rater agreement among triage nurses is likely to improve.

However, there was a slight decrease in the ED LoS before and after PBL, although the decrease was not statistically significant. This was different from previous studies [5, 6] showing that the ED LoS was reduced due to accurate patient classification. In the study of Jobé, Ghuysen, & D’Orio, accurate patient classification helped reduce the ED LoS for patients who came to the ED for chest pain [6]. Direct comparisons, not pre-post comparisons, were difficult. However, Erimsah, Yaka, Yilmaz, Kama, & Pekdemir and Kim et al. have reported that adequate patient classification can help reduce the ED LoS [34, 35]. Pierce & Gormley have also reported that primary and secondary assessments can improve the classification of emergency patients, resulting in a shorter ED LoS as a result of applying a more accurate and appropriate patient classification system [36]. In the present study, the ED LoS was reduced approximately 30 min after the PBL meeting, although such reduction was not statistically significant. Various factors may exist to explain the reduction in the ED LoS. It would be hard to say that the improvement in the consistency of KTAS classification only affected the reduction in the ED LoS. Further studies will be needed to determine whether the agreement of emergency severity classification affects the ED LoS.

### Limitations

This study has some limitations. First, the inter-rater agreement of KTAS classification might have improved as nurses’ experiences in patient classification work increased after PBL meeting compared to those before PBL meeting. Second, the participation rate was very high, although PBL meeting for nurses was voluntary. However, there were also a small number of nurses who showed low participation frequency. For nurses who did not participate in the problem based-learning meeting, results of the meeting were shared using SNS ‘Band’ application, ‘Groupware email’, and ‘Shared Note’. Third, in this study, the inter-rater agreement between triage nurses and Gold Standard nurses was evaluated. However, we did not determine agreements among the three evaluators. Such information might provide more accurate classification results. Fourth, although most of the nurses who participated in the PBL before and after meeting were the same, the before and after were not completely the same because there were two nurses who resigned and three nurses who were newly starting the triage task. Lastly, this study was a single-center study conducted at a general hospital in Seoul. Thus, it is difficult to generalize results of this study to other settings.

## Conclusions

In this study, PBL meeting for triage nurses improved the inter-rater agreement of KTAS classification results and the self-efficacy of triage nurses for emergency patient severity classification. Therefore, PBL meeting led by triage nurses can contribute to patient safety in hospitals by enhancing the expertise of triage nurses and increasing the accuracy of triage classification. Further studies should be conducted to determine whether the agreement of KTAS classification results among triage nurses affects the ED LoS. Educational programs need to be developed to enhance the capacity and professionalism of triage nurses.

## Data Availability

The datasets used and/or analysed during the current study are available from the corresponding author upon reasonable request.

## Abbreviations

ED: emergency department
KTAS: Korean Triage and Acuity Scale
CTAS: Canadian Triage and Acuity Scale
PBL: problem-based learning
EMR: electronic medical record
ED LoS: length of emergency department stay
